# No difference in stability among various knee flexion angles during fixation of anterolateral ligament reconstruction or lateral extra‐articular tenodesis: A systematic review and meta‐analysis of biomechanical studies

**DOI:** 10.1002/jeo2.12079

**Published:** 2024-07-15

**Authors:** David A. Kolin, Ruth H. Jones, Benton E. Heyworth, Bridget Jivanelli, SATURN Study Group, Peter D. Fabricant

**Affiliations:** ^1^ Orthopedic Surgery Department New York New York USA; ^2^ Pediatric Orthopedic Surgery Service New York New York USA; ^3^ Orthopedics and Sports Medicine Department Boston Children's Hospital Boston Massachusetts USA; ^4^ Kim Barrett Memorial Library New York New York USA

**Keywords:** anterior cruciate ligament, anterolateral ligament reconstruction, anterior translation, flexion angle, internal rotation, lateral extra‐articular tenodesis

## Abstract

**Purpose:**

The purpose of this study was to investigate the effect of anterolateral ligament reconstruction (ALLR) or lateral extra‐articular tenodesis (LET) fixation at low versus high flexion angles during anterior cruciate ligament reconstruction (ACLR) on rotation or translational knee stability.

**Methods:**

The inclusion criteria for this study were (1) cadaveric study, (2) cadaveric specimens underwent ACLR, (3) cadaveric specimen underwent ALLR or LET and (4) specimen preparation technique described the knee flexion angle at the time of ALLR or LET tensioning and fixation. A priori, ‘low flexion’ was defined as 0–30° and ‘high flexion’ was defined as 60–90° at graft fixation. Main outcomes of interest included internal rotation and anterior translation.

**Results:**

Data from 92 cadaveric knees (from 9 studies) were extracted and included in the meta‐analysis. The mean pooled value for internal rotation was 10.1° (95% confidence interval [CI], 5.7–14.5°) for the low flexion group and 11.5° (95% CI, 7.4–15.7°) for the high flexion group (n.s.). The mean pooled value for anterior translation was 4.3 mm (95% CI, 0.5–8.1 mm) for the low flexion group and 3.0 mm (95% CI, 1.1–5.0 mm) for the high flexion group (n.s.).

**Conclusion:**

This meta‐analysis of existing biomechanical research found that the rotational and translational stability of the knee were not significantly different between scenarios in which ALLR or LET fixation was performed at low knee flexion angles (0–30°) versus high knee flexion angles (60–90°).

**Level of Evidence:**

Level IV.

**Abbreviations**
ACLR: anterior cruciate ligament reconstruction
ALL: anterolateral ligament
ALLR: anterolateral ligament reconstruction
AT: anterior translation
IKDC: International Knee Documentation Committee
IR: internal rotation
LET: lateral extra‐articular tenodesis

## INTRODUCTION

Anterior cruciate ligament reconstruction (ACLR) is being supplemented with anterolateral ligament (ALL) reconstruction (ALLR) or lateral extra‐articular tenodesis (LET) with increasing frequency [26]. Both ALLR and LET have seen a resurgence in recent years as an adjunct to ACLR due to their potential to mitigate risk of graft rupture or laxity after ACLR [19]. Because the ALL runs in the anteroinferior direction from the lateral femoral epicondyle to the anterolateral aspect of the proximal tibia, it is hypothesised to play a role in both translational and rotational knee stabilisation [28]. However, there is also concern that ALLR and LET may contribute to overconstraint of the lateral compartment and accelerate degenerative changes [10, 13, 25]. A laboratory study by Neri et al. found that lateral tibiofemoral contact pressures were greater with a variety of lateral extraarticular augmentation procedures, with increased contact pressures causing concern for potential acceleration of degenerative joint disease [22].

Several conflicting in vivo studies have investigated the differences in ACLR surgery with or without ALLR or LET procedures. Compared to isolated ACLR, several studies have found a lower risk of complications and re‐surgery and a higher chance of returning to sport when ACLR is accompanied by ALLR [1, 21, 29, 33]. Some studies have also found that ALLR and LET have resulted in superior knee laxity relative to isolated ACLR, but did not improve other objective or subjective outcomes [11, 12]. Other studies suggest that International Knee Documentation Committee scores are similar between patients who underwent isolated ACLR and those who underwent combined procedures [3, 27, 32].

Given the variety of outcomes associated with the combined procedure, intraoperative differences have been investigated in the context of ALLR and LET, including the knee flexion angle at the time of fixation. Fixation angles range from 0° to 90°, with no clear consensus on the most optimal angle at the time of fixation [16]. In this study, we conducted a systematic review and meta‐analysis of existing biomechanical cadaveric studies to determine if the knee flexion angle at the time of ALLR or LET fixation affected any measured biomechanical outcomes that act as proxies for stability, including internal rotation and anterior translation.

## METHODS

A literature search was conducted by a medical librarian coinvestigator (B. J.) using PubMed, Embase and Cochrane Library. Keywords for the search included [‘IT band’, ‘iliotibial band’, ‘anterolateral ligament’, ‘lateral extra‐articular tenodesis’] AND [‘anterior cruciate ligament’ OR ‘anterior cruciate ligament reconstruction’] along with associated MeSH terms. The full search strategy can be found in (Appendix S1). Publications were reviewed starting from 1 January 2001. The Covidence systematic review software package was utilised to assist with assessing de‐duplication of references, abstract screening and screening full text. All abstracts and manuscripts were manually screened.

### Eligibility criteria

**Figure 1:**
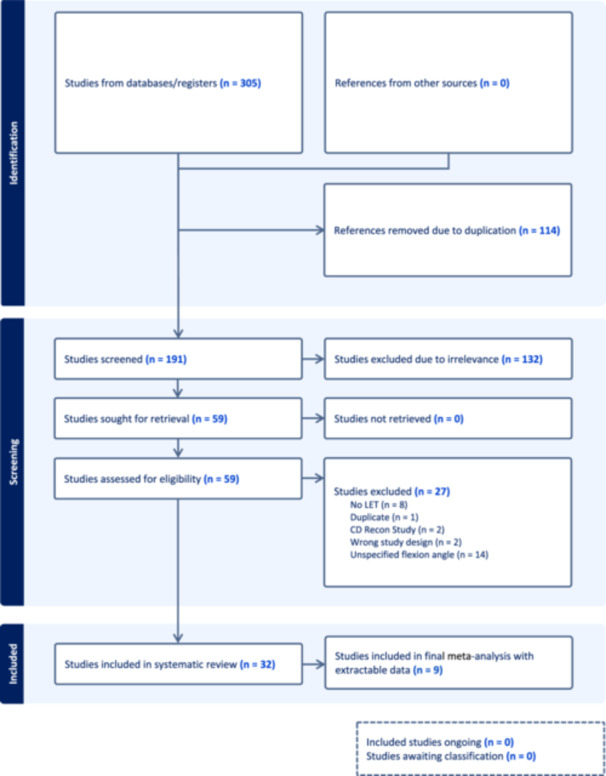
Preferred Reporting Items for Systematic Reviews and Meta‐analyses study selection flow diagram.

The inclusion criteria for this systematic review were (1) cadaveric study, (2) cadaveric specimens underwent ACLR, (3) cadaveric specimens underwent ALLR or LET, and (4) specimen preparation technique described the knee flexion angle at the time of ALLR or LET tensioning and fixation. To be included in the meta‐analysis, studies also must have reported biomechanical analysis of internal rotation and anterior translation. Exclusion criteria were systematic reviews, meta‐analyses, trial protocols, surgical technique papers, articles that failed to report knee flexion angle at the time of ALLR/LET fixation, or articles with intermediate (31–59°) knee flexion angles at the time of fixation (Figure 1). Intermediate flexion angles at fixation were excluded because they constituted neither low nor high flexion angles. Including them in an analysis of high versus low flexion angle at fixation would have biased the results of the study toward the null and increased the risk of Type II error.

### Demographics and outcomes

Demographic measures of interest included the number of knees in each study. Main outcomes of interest included (1) internal rotation and (2) anterior translation. Each main outcome was assessed both overall and at four different angles of flexion: 0°, 30°, 60° and 90°. Subgroup analyses of interest investigated outcomes for ALLR and LET separately.

### Statistical analysis

The number of cadaveric knees in each study was recorded. Pooled means for continuous outcomes including internal rotation and anterior translation were calculated using weighted means with a random‐effects model, using a restricted maximum‐likelihood estimator. Independent samples *t*‐tests using weighted values were used to compare results between the low and high flexion groups. The two‐tailed significance threshold was set to *p* ≤ 0.05. All analyses were performed with the use of R software, version 4.0 (R Foundation for Statistical Computing).

## RESULTS

The meta‐analysis involved pooling data from 92 cadaveric knees (Table 1). There were six studies (36 knees) contributing to the low flexion fixation group and seven studies (46 knees) contributing to the high flexion fixation group. In the study performed by Schon et al. [30], all 10 knees were tested with both low and high flexion ALLR fixation. Seven studies examined the use of ALLR and six studies examined the use of LET. There were four studies that explored the use of low and high flexion angles at the time of fixation and four studies that explored both the use of ALLR LET.

**Table 1:** ALLR/LET fixation studies.

References	ALLR or LET	Flexion angle at ALLR/LET fixation (°)	Total cadaveric knees
Jette et al. [15]	ALLR and LET	0	12
Neri et al. [23]	LET	30	10
Bonanzinga et al. [4]	ALLR	70	10
Nitri et al. [24]	ALLR	75	10
Delaloye et al. [6]	ALLR and LET	0 (ALLR) and 70 (LET)	6
Smith et al. [31]	ALLR and LET	0 (ALLR) and 70 (LET)	12
Geeslin et al. [9]	ALLR and LET	30 and 70 (randomised for ALLR and LET)	10
Schon et al. [30]	ALLR	0, 15, 30, 45, 60, 75 and 90	10
Lagae et al. [18]	LET	60	12

Abbreviations: ALLR, anterolateral ligament reconstruction; LET, lateral extra‐articular tenodesis.

### Main outcomes

The main outcomes for this study were internal rotation and anterior translation for knees fixed at low and high flexion angles. The mean pooled value for internal rotation was 10.1° (95% confidence interval [CI], 5.7–14.5°) for the low flexion group and 11.5° (95% CI, 7.4–15.7°) for the high flexion group (n.s.). The mean pooled value for anterior translation was 4.3 mm (95% CI, 0.5–8.1 mm) for the low flexion group and 3.0 mm (95% CI, 1.1–5.0 mm) for the high flexion group (n.s.).

### ALLR versus LET

Internal rotation and anterior translation pooled values were calculated for ALLR and LET in the low and high flexion fixation groups at four degrees of flexion tested: 0°, 30°, 60° and 90° (Table 2). For ALLR and LET at low flexion, internal rotation values were 9.6° (95% CI, 3.9–15.4°) and 11.5° (95% CI, 5.6–17.5°), respectively (n.s.). At high flexion, internal rotation was 13.5° (95% CI, 12.2–14.7°) for ALLR and 8.6° (95% CI, −8.4 to 25.6°) for LET (n.s.). Anterior translation was 3.2 mm (95% CI, −2.4 to 8.8 mm) for ALLR and 6.0 mm (95% CI, 0.0–11.9 mm) for LET at low flexion (n.s.) and was 3.6 mm (95% CI, 2.4–4.8 mm) for ALLR and 2.6 mm (95% CI, −0.8 to 6.1 mm) for LET at high flexion (n.s.).

**Table 2:** ALLR and LET pooled internal rotation and anterior translation at 0°, 30°, 60° and 90° of flexion.

Degrees	ALLR				LET			
	Low flexion		High flexion		Low flexion		High flexion	
	IR	AT	IR	AT	IR	AT	IR	AT
0°	7.4 (2.8–12.0)	2.7 (−2.1 to 7.5)	9.6 (8.6–10.5)	4.2 (3.8–4.6)	7.1 (−6.1 to 20.2)	7.7 (6.0–9.4)	4.7 (−5.7 to 15.1)	2.3 (−1.4 to 6.0)
30°	11.9 (5.0–18.8)	4.3 (−2.4 to 10.9)	16.2 (14.8–17.6)	6.6 (3.0–10.3)	13.9 (4.6–23.1)	9.6 (7.4–11.9)	9.2 (−9.6 to 28.0)	3.2 (−1.6 to 8.0)
60°	11.9 (4.2–19.5)	5.0 (−4.0 to 14.0)	15.7 (14.4–17.1)	4.4 (3.7–5.1)	12.7 (10.2–15.2)	9.7 (8.3–11.1)	11.0 (−9.2 to 31.2)	5.4 (3.1–7.7)
90°	8.7 (3.5–13.9)	2.5 (−2.3 to 7.3)	12.3 (11.2–13.5)	3.4 (2.8–4.0)	12.4 (11.3–13.6)	5.6 (1.2–10.1)	9.5 (−9.2 to 28.1)	1.6 (−1.0 to 4.2)

Abbreviations: ALLR, anterolateral ligament reconstruction; AT, anterior translation; IR, internal rotation; LET, lateral extra‐articular tenodesis.

## DISCUSSION

The most important finding of this study was that fixation at low flexion angles did not appear to lead to any relevant differences in internal rotation or anterior translation when compared to fixation at high flexion angles. Furthermore, comparisons between ALLR and LET identified no differences in internal rotation nor anterior translation measures.

The primary function of the ACL is to control tibial anterior translation and secondarily to limit internal rotation [7, 17]. It is thought that residual rotational laxity following ACLR has a detrimental effect on clinical outcomes, including patient satisfaction and eventual return to sport [5, 8]. For this reason, choice of ACL autograft has been widely investigated. However, no major differences have been demonstrated. Similarly, the addition of ALLR or LET to ACLR has been explored as a potential benefit to ACLR biomechanical outcomes. The ALL acts as a secondary stabiliser to the ACL, adjunctively aiding in decreasing or eliminating any excessive anterior tibial translation, internal tibial rotation and knee pivot shift. Therefore, its role in clinical scenarios of ACL failure and failure of ACL reconstruction grafts is being explored in great detail [2]. However, some authors contest that the ALL plays a negligible role in physiologic ranges of tibial translation [20]. The LET procedure has also been shown to enhance anterolateral knee stability, reduce laxity and pivot shift and decrease risk of ACL graft failure by fixing an iliotibial band graft to the femur. While both ALLR and LET, in addition to traditional ACLR, have the potential to reduce tibiofemoral motion and internal rotation, LET possibly leads to a greater reduction in isolated internal rotation than ALLR [9]. In the context of investigating varying knee flexion angles for ALLR or LET, no known optimal or superior technique has been determined in prior studies. Similarly, the current study was unable to identify any differences in biomechanical outcomes, such as internal rotation or translation, between procedures performed at low or high flexion angles at the time of fixation and there were no differences between ALLR or LET.

Additionally, multiple clinical studies have investigated differences between ALLR and LET on patient outcomes. A systematic review of patients undergoing ALLR or LET identified that LET could lead to worse anterior instability than ALLR [26]. However, the authors found that rotational stability and patient‐reported outcome measures were similar between the two techniques. A separate systematic review found that, in contrast to ALLR, the LET procedure was associated with greater stiffness [21]. Those authors postulated that the finding may be due to the fact that the LET procedure involves a nonanatomic reconstruction that could overconstrain the knee. To the contrary, the current study found no evidence of differences in rotational stability between ALLR and LET in this cadaveric systematic review and meta‐analysis.

There are important limitations to note with this study. First, there were several constituent studies identified in this review that investigated ALLR and LET at different fixation angles that did not have values available for extraction, as they were reported in figures with no discrete values noted (e.g., in tables). For some articles, authors were able to provide tables of values, not noted within manuscript text; however, for other studies, authors were unable to provide tabulated outcome data for analysis despite pertinent data present within figures. Although this nondifferential data loss is unlikely to bias the results of the current study, it does decrease the number of specimens that can be included in the analysis, thus reducing statistical power to detect any differences. Second, while internal rotation and anterior translation are important outcomes, there were a number of outcomes that could not be assessed in this systematic review and meta‐analysis. Perhaps most notably, cadaveric studies cannot reliably assess ACL graft failure rates, arguably the most important clinical outcome. Contact pressures could not be reliably analysed in this study, due to limited reporting in the constituent studies. Only one study by Neri et al. reported contact pressures in a manner suitable for meta‐analysis extraction [22]. In their study, they found that the addition of ALLR or the modified Ellison procedure did not change overall lateral tibiofemoral contact pressures from 0° to 90° of knee flexion, while the Lemaire and Macintosh procedures increased contact pressures. Inderhaug et al. found that risk of overconstraint was low when the tibia was positioned in neutral rotation at the time of fixation and Novaretti et al. similarly found that LET with a semitendinosus graft did not significantly increase pressure in the lateral compartment [14, 25]. In contrast, Marom et al. found significantly increased anterolateral tibial plateau contact pressures with LET [19]. Third, only nine studies were able to be included in this meta‐analysis, which is relatively low. Future studies that assess the association of ALLR and LET fixation angles with clinical outcomes may be able to identify important biomechanical outcome differences with increased statistical power as a result of larger sample sizes.

## CONCLUSION

In conclusion, this meta‐analysis of existing biomechanical research found that the rotational and anterior translational stability of the knee were not significantly different between scenarios in which ALLR or LET fixation was performed at low knee flexion angles (0–30°) versus high knee flexion angles (60–90°). These data suggest that from a biomechanical perspective, in the absence of evidence demonstrating a clinically superior technique, either technique preference is reasonable depending on surgeon preference. Clearly, future research is needed to quantify contact pressures, graft failure rates and other parameters designed to elucidate the optimal technique for different patients undergoing this increasingly common category of procedures.

## SATURN Study Group

Benton E. Heyworth, MD, FAAOS, Yi‐Meng Yen, MD, PhD, FAAOS, Dennis E. Kramer, MD, FAAOS, Mininder S. Kocher, MD, MPH, FAAOS, Peter D. Fabricant, MD, MPH, FAAOS, Andrew Tennant Pennock, MD, FAAOS, Jeffrey J. Nepple, MD, FAAOS, Samuel Clifton Willimon, MD, FAAOS, Crystal Ann Perkins, MD, FAAOS, Henry Bone Ellis Jr, MD, FAAOS, Philip L. Wilson, MD, FAAOS, Michael McClincy, MD, James Everett Voos, MD, FAAOS, David D. Spence, MD, FAAOS.

## AUTHOR CONTRIBUTIONS

**David A. Kolin**: Visualisation; methodology; data curation; formal analysis; writing—original draft; writing—reviewing and editing. **Ruth H. Jones**: Study coordination; writing—original draft; writing—reviewing and editing. **Benton E. Heyworth**: Visualisation; writing—reviewing and editing. **Bridget Jivanlli**: Methodology; data curation; writing—reviewing and editing. **SATURN Study Group**: Visualisation. **Peter D. Fabricant**: Visualisation; writing—reviewing and editing; supervision.

## CONFLICTS OF INTEREST STATEMENT

David A. Kolin: Editorial or Governing Boards: Associate Editor: Clinical Orthopedics and Related Research (CORR); Reveiwer: *Journal of Bone & Joint Surgery* (JBJS). Benton E. Heyworth: Allosource: Other financial or material support; Imagen Technologies, Inc.: Stock or stock Options; Pediatric Orthopaedic Society of North America: Board or committee member; Pediatric Research in Sports Medicine (PRISM): Board or committee member; Springer: Publishing royalties, financial or material support; Vericel: Other financial or material support. Peter D. Fabricant: Editorial or Governing Boards: Associate Editor: Clinical Orthopedics and Related Research (CORR); Consultant: BICMD, Inc.; Ownership Interest: OssoVR; HS2, LLC; HSS ASC Development Network, LLC; Joint Effort Administrative Services Organization, LLC. The remaining authors declare no conflict of interest.

## ETHICS STATEMENT

This study was determined to be exempt from Institutional Review Board approval.

## Supporting information

Supporting information.

## Data Availability

The data that support the findings of this study are available on request from the corresponding author. The data are not publicly available due to privacy or ethical restrictions.
