# Supplementary material for: No difference in stability among various knee flexion angles during fixation of anterolateral ligament reconstruction or lateral extra‐articular tenodesis: A systematic review and meta‐analysis of biomechanical studies
Source: J Exp Orthop. 2024 Jul 15;11(3):e12079. doi: 10.1002/jeo2.12079 (PMC11250156; doi:10.1002/jeo2.12079)
Supplement: Supplementary file 1 — Supporting information. [file JEO2-11-e12079-s001.docx]

**Appendix 1.** Complete Search Strategy

**PubMed**

(2001:2021[pdat])
AND
(IT band[tw] OR Iliotibial band[tw] OR anterolateral ligament[tw] OR ALL reconstruction[tw] OR lateral extra-articular tenodesis[tw] OR LET[tw])
AND
(anterior cruciate ligament[tw] OR "Anterior Cruciate Ligament"[Mesh] OR anterior cruciate ligament reconstruction[tw] OR "Anterior Cruciate Ligament Reconstruction"[Mesh])
AND
(Cadaveric[tw] OR "Cadaver"[Mesh] OR cadaver[tw])

**EMBASE**

(2001:2021[pdat])
AND
("IT band":ti,ab,de,tn,kw OR "Iliotibial band":ti,ab,de,tn,kw OR "anterolateral ligament":ti,ab,de,tn,kw OR "ALL reconstruction":ti,ab,de,tn,kw OR "lateral extra-articular tenodesis":ti,ab,de,tn,kw OR LET:ti,ab,de,tn,kw)
AND
("anterior cruciate ligament":ti,ab,de,tn,kw OR 'Anterior Cruciate Ligament'/exp OR "anterior cruciate ligament reconstruction":ti,ab,de,tn,kw OR 'Anterior Cruciate Ligament Reconstruction'/exp)
AND
(Cadaveric:ti,ab,de,tn,kw OR 'Cadaver'/exp OR cadaver:ti,ab,de,tn,kw)

**Cochrane**

(2001:2021[pdat])
AND
("IT band":ti,ab,kw OR "Iliotibial band":ti,ab,kw OR "anterolateral ligament":ti,ab,kw OR "ALL reconstruction":ti,ab,kw OR "lateral extra-articular tenodesis":ti,ab,kw OR LET:ti,ab,kw)
AND
("anterior cruciate ligament":ti,ab,kw OR [mh "Anterior Cruciate Ligament"] OR "anterior cruciate ligament reconstruction":ti,ab,kw OR [mh "Anterior Cruciate Ligament Reconstruction"])
AND
(Cadaveric:ti,ab,kw OR [mh Cadaver] OR cadaver:ti,ab,kw)
